# Effects of Glucocorticoids on the Inner Ear

**DOI:** 10.3389/fsurg.2020.596383

**Published:** 2021-01-11

**Authors:** Taizo Takeda, Setsuko Takeda, Akinobu Kakigi

**Affiliations:** ^1^Kochi Medical School, Kochi, Japan; ^2^Department of Otorhinolaryngology Head and Neck Surgery, Graduate School of Medicine, Kobe University, Kobe, Japan

**Keywords:** hydrocortisone, dexamehtasone, endolymphatic hydrops (EH), sudden deafness, Meniere's disease, steroid therapy

## Abstract

**Hypothesis:** Recently, several lines of evidence have suggested that the inner ear is under hormonal control. It is likely that steroids have some influence on the inner ear.

**Background:** Many clinicians have been empirically using steroids for the treatment of diseases associated with endolymphatic hydrops. The theoretical grounds for this are not clear, and there have been a number of debates on the effectiveness of steroid treatment. Furthermore, there are few reports on histological observations of the influences of steroids on the cochlea.

**Method:** Fifteen guinea pigs (30 ears) were divided into three groups. In the control group, physiological saline solution was administered intra-peritoneally for 3 days. In two steroid groups, 40 mg/kg/day of hydrocortisone or 4 mg/kg/day of dexamethasone was administered intra-peritoneally for 3 days. Extension of Reissner's membrane and volume change of the scala media were checked 6 h after the last administration. The degree of Reissner's membrane extension and volumetric change of the scala media were quantitatively measured with the use of a video-digitizer.

**Results:** We did not identify any distinct changes in the cochlea of the control group. In contrast, the extension of Reissner's membrane and endolymphatic hydrops were observed in the animals in the steroid groups. Statistical analysis revealed that Reissner's membrane extended significantly in the steroid groups, and that the volume of the scala media also increased significantly.

**Conclusion:** This is the first report to investigate the effects of systemic administration of glucocorticoids on guineapig cochlea. The extension of Reissner's membrane and dilated endolymphatic space were evident in the steroid groups. However, the underlying mechanism of histological changes was not clear, marked care needs to be taken when administering steroids to patients with Meniere's disease whose histological feature is endolymphatic hydrops.

## Introduction

An allergic reaction had been suspected to be one of the contributing factors to Meniere's disease. Based on the allergy theory, Hauser (1) treated two cases of Meniere's disease with corticosteroids in 1959 and obtained favorable results. However, steroid therapy received little attention, and its use did not progress markedly in the treatment of Meniere's disease (2). Also, serologic test results did not support the allergy theory (3). In 1986, Beck (4) concluded that corticosteroid therapy, which is very effective for allergic diseases, had no marked effect against Meniere's disease. Based on this result, he did not support the allergy theory of Meniere's disease. Additionally, Stahle (2) also disagreed with the allergy theory. Since that time, there have been few advances and discussions regarding steroid therapy based on allergy.

Since McCabe (5) proposed the new concept of “autoimmune sensorineural hearing loss,” it has been highlighted that some cases of Meniere's disease might be caused by autoimmune factors. This concept encouraged the use of steroids in the treatment of Meniere's disease. Shea (6) reported that about 10% of patients with typical Meniere's disease showed improved hearing and decreased dizziness following dexamethasone treatment. Further, Shea Jr. (7) achieved better results by administering dexamethasone by perfusion *via* the round window plus intravenously. In contrast, Silverstein (8) obtained different results from a double-blind, crossover trial of dexamethasone inner ear perfusion. Intratympanic administration of dexamethasone to patients with Meniere's disease led to no benefit over a placebo for the treatment of hearing loss and tinnitus.

As evident in the literature, corticosteroids have often been used for the treatment of Meniere's disease. However, many clinical trials failed to show the conclusive efficacy of steroid therapy regardless of whether steroid was administered systemically or intratympanically (9–12). Further, the rationality for this use is still controversial. In fact, there is no experimental evidence that this treatment is beneficial. In the present study, we administered hydrocortisone and dexamethasone serially to normal guinea pigs and investigated the histological changes in the inner ear.

## Materials and Methods

Fifteen Hartley guinea pigs with a positive Preyer's reflex, weighing between 300 g and 400 g, were used.

*Experimental animals*: Fifteen guinea pigs were divided into three groups (Table 1): Control group: five animals (10 ears) were administered physiological saline aqueous solution (0.2 ml) intra-peritoneally twice a day for 3 days. Steroid group: this group was composed of two groups, hydrocortisone and dexamethasone groups. The hydrocortisone group (5 animals, 10 ears) were administered 20 mg/kg of hydrocortisone intra-peritoneally. Hydrocortisone was also given twice a day for 3 days. Twenty mg/kg of hydrocortisone was reported to be an effective dose (ED50) for anti-inflammatory activity in rats (13). The dexamethasone group (5 animals, 10 ears) was intra-peritoneally administered 2 mg/kg of dexamethasone twice a day for 3 days. The effective dose (ED50) for anti-anaphylaxis activity in rats was reported to be 1.8 mg/kg (14). All animals were sacrificed 6 h after the last administration to observe histological changes in Reissner's membrane.

**Table 1:** Extension ratios of Reissner's membrane (ER-R) (%) and increase ratios of the cross-sectional area of the scala media (IR-S) (%) of control and steroid groups.

	**Control**	**Hydrocortisone**	**Dexamethasone**
	**(*n* = 10)**	**(*n* = 10)**	**(*n* = 10)**
ER-R (%)	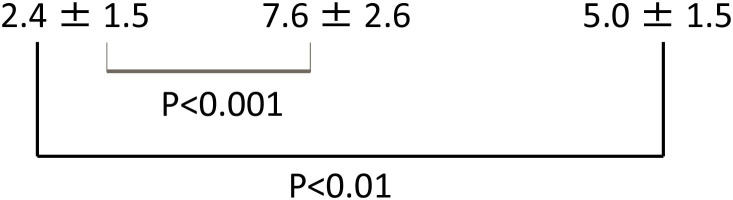		
IR-S (%)	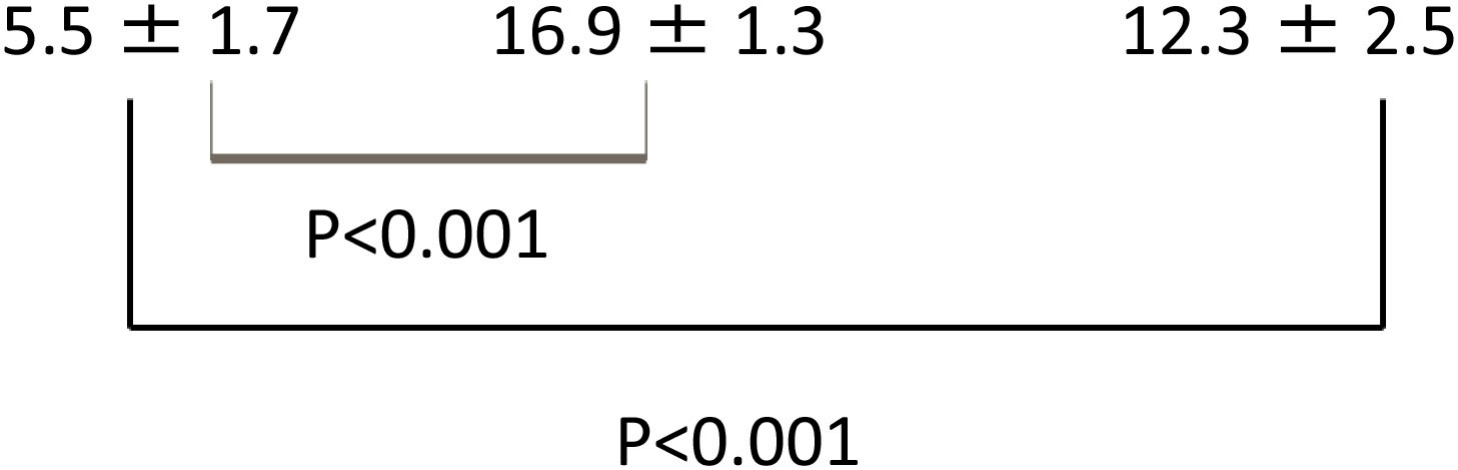		

*Both ER-R and IR-S of the steroid groups were significantly higher than those of the control group using Student's t-test*.

*Preparation procedure*: We obtained both sides of the temporal bones immediately following fixation with perfusion of 10% formalin solution under deep anesthesia with intra-peritoneal injections of pentobarbital. We kept the temporal bones in 10% formalin solution for 10 days or more. They were decalcified with 5% trichloroacetic acid and dehydrated with alcohol in increasing concentrations, and were then embedded in paraffin and celloidin. The prepared blocks were cut into 6 μm horizontal sections. The sections were stained with hematoxylin and eosin and studied under a light microscope.

**Figure 1:**
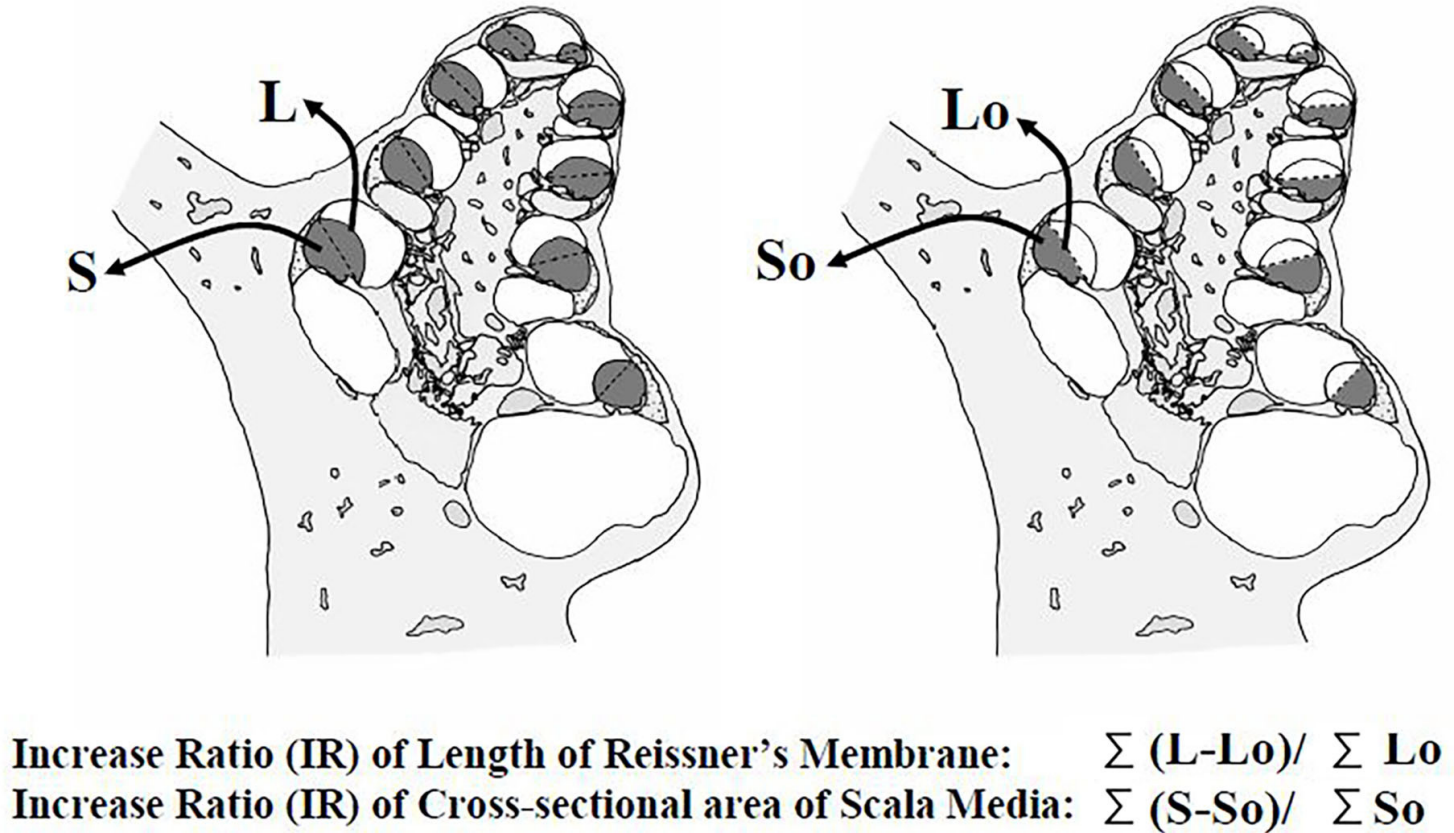
Four parameters for assessment of the extension of Reissner's membrane and dilation of the scala media. The length of the extended Reissner's membrane (L), the cross-sectional area of the bulging scala media (dotted area, S), the reference length of Reissner's membrane (Lo); the length between the points of attachment of the stria vascularis and of attachment of the basilar membrane, and the cross-sectional area of the reference scala media (So), enclosed by the reference Reissner's membrane. Extension ratios of Reissner's membrane and increase ratios of the scala media were calculated from these data.

*Measurement procedure*: The extension of Reissner's membrane and increase of the endolymphatic space were quantitatively analyzed in addition to conventional observations of the morphological changes in the cochlea and endolymphatic sac. For this analysis, the following four parameters were measured (Figure 1) from the mid-modiolar sections of the cochlea: (1) the length of the extended Reissner's membrane (L), (2) the cross-sectional area of the bulged scala media (dotted area, S), (3) the reference length of Reissner's membrane (Lo), and (4) the cross-sectional area of the reference space of the scala media (So). The measurement system was composed of a video camera, a computer, and a digitizer (Video Micro Meter VM-30, Olympus Co., Tokyo, Japan). The second author measured those parameters without any notice about which specimen is which group.

From these parameters measured at all turns, the extension ratios (%) of Reissner's membrane and increase ratios (%) of the cross-sectional area of the scala media were calculated according to the formula described below:

Extension ratio of Reissner's membrane (ER-R) (%) = 100 × (ΣL–ΣL0)/ΣL0

Increase ratio of the cross-sectional area of the scala media (IR-S) (%) = 100 × (ΣS–ΣS0)/ΣS0

Σ means the summation of the values of four turns.

This study was approved by the Kochi Medical School Animal Care and Use Committee (Approval No. B-00094) and conformed to the Animal Welfare Act and the guiding principles for animal care produced by the Ministry of Education, Culture, Sports, Science and Technology, Japan.

## Results

**Figure 2:**
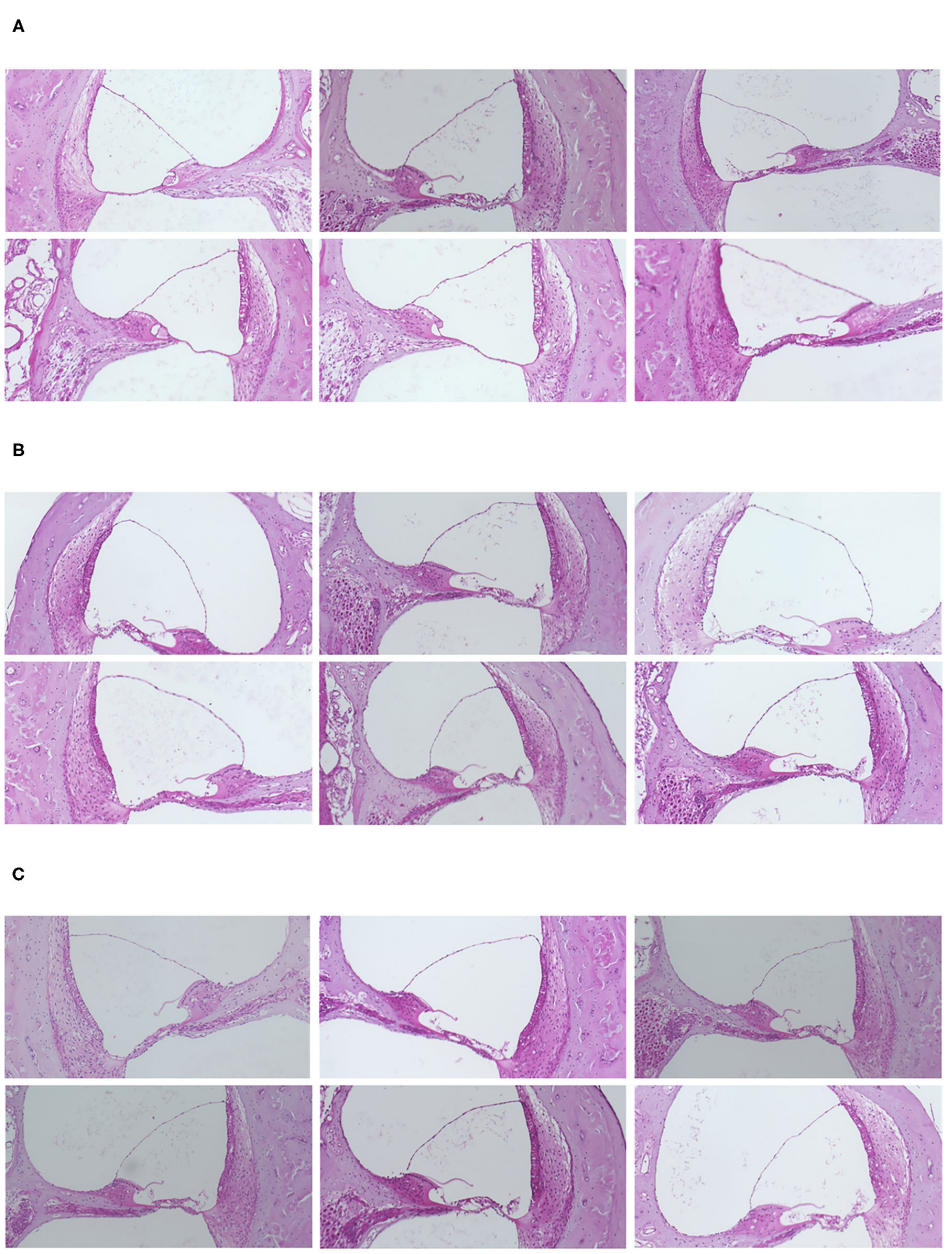
**(A)** Representative histological findings in the normal and steroid groups. Slight but distinct endolymphatic hydrops was observed in the steroid groups. **(A)** Control group. **(B)** Hydrocortisone group. **(C)** Dexamethasone group.

Reissner's membranes were extended and slight endolymphatic hydrops was found in 17 (85.0 %) of 20 ears following the administration of hydrocortisone. Figure 2 shows representative histological findings from normal and steroid groups. The extension of Reissner's membrane and dilated endolymphatic space were evident in the steroid groups.

Extension ratios of Reissner's membrane (ER-R) (%) and increase ratios of the cross-sectional area of the scala media (IR-S) (%) of the 3 groups (Mean ± S.D.) are shown in Table 1. These three sample data were considered to come from a population with a normal distribution, hence the null hypothesis of normality assumption was rejected by the Kolmogorov-Smirnov normality test (*p* > 0.05). Thus, the *t*-test was applied for the statistical analysis of these data. ER-R and IR-S of the hydrocortisone group were significantly higher than those of the control group (*P* < 0.001, *t*-test), and ER-R and IR-S of the glucocorticoid group were also significantly higher than those of the control group (*P* < 0.001, *t*-test). Statistical analysis was performed with the use of the commercially available software StatMate V (ATMS Co. Tokyo, Japan).

Steroid administration led to no marked histological change other than the volumetric changes of the scala media. Neither vacuolization nor atrophic change was noted in the stria vascularis or vestibular sensory epithelium. The endolymphatic sac also had a normal appearance light-microscopically. No abnormal infiltration was observed in the lumen of the endolymphatic sac.

## Discussion

This is the first report to investigate the effects of systemic administration of glucocorticoids on guineapig cochlea. The extension of Reissner's membrane and dilated endolymphatic space were evident in the steroid groups. Recently, receptors of gluco- and mineralocolcicoid hormones were detected in the inner ear by different techniques (15, 16). A more detailed investigation by an Enzyme Linked Immuno-Sorbent Assay (ELISA) revealed that glucocorticoid receptors were distributed not only in the spiral ligament but also in the stria vascularis, although at lower levels (17). The localization of glucocorticoid receptors was also confirmed by *in situ* hybridization histochemical studies (18). Furthermore, the expression of mineralocorticoid type I receptor mRNA was also demonstrated in marginal cells of the stria vascularis by *in situ* hybridization histochemistry (19). These facts support the suggestion that adrenocorticoids play a biological role in the inner ear function.

It is worthy of attention that adrenal steroid receptors are present in the stria vascularis, which is considered to participate in ion and fluid homeostasis in the inner ear. Although we have little information available on the mechanism of action of these steroid hormones in the inner ear, it is likely that the excess of adrenal steroids might have some influence on ion and fluid homeostasis in the inner ear. In support of this, it has been reported that endolymphatic hydrops was induced with aldosterone (20).

In the present study, serial administration of the synthetic glucocorticoid hydrocortisone also resulted in the extension of Reissner's membrane and slight endolymphatic hydrops. The stria vascularis and spiral ligament are well-known to contain varying levels of Na, K-ATPase, which is considered to be an important enzyme involved in ion and fluid transport (21–23). Elevated serum levels of glucocorticoid have been reported to be correlated with significant increases in Na, K-ATPase α-subunit levels, both in the stria vascularis and spiral ligament (24). This increase in the activity of Na, K-ATPase is likely to be one of the possible factors contributing to the steroid-induced hydrops formation observed in the present study. Another possible factor is steroid-induced upregulation of aquaporin 3 (25, 26). Aquaporins are known to play roles in the homeostatic mechanism regulating water and ionic balance in the inner ear fluids (27, 28). Therefore, the present hydrops formation may have resulted from the acceleration of endolymphatic water homeostasis via glucocorticoid-enhanced AQP3 in the inner ear.

Although no morphologic changes except extension of Reissner's membrane were observed in the present study under a light microscope, there is some evidence that changes in levels of circulating adrenal steroids influence the inner ear ultra-structurally as well as functionally (29–32). An increase in the intercellular space and a decrease in basolateral infoldings in the stria vascularis and dark cells of the vestibular epithelium were observed in adrenalectomized animals (29, 30). Furthermore, the restoration of circulating adrenocorticosteroids levels resulted in recovery of the cellular architecture of the stria vascularis and dark cells of the vestibular epithelium in adrenalectomized animals (31, 32). These findings provide indirect evidence that adrenal steroids are involved in the cellular regulations of inner ear tissues concerned with fluid and ionic microhomeostasis.

As mentioned above, it has been reported that under an abnormal state of adrenal steroids, ultrastructural changes in sensory and non-sensory cells of the cochlea and vestibular organs, although small, have been detected (33). It is unknown whether these changes are pathologic. Regarding functional aspects of the inner ear, however, glucocorticoid intake was reported to induce an elevated threshold of human auditory evoked responses (34, 35).

Glucocorticoids with a strong anti-inflammatory action have been prescribed empirically for Meniere's disease (1, 6–12). It is unknown how many dosages in a human correspond to the 20 mg/kg of hydrocortisone and 2 mg/kg of dexamethasone used in the present study. The dosage is the effective dose (ED50) for anti-inflammatory action or anti-anaphylaxis action in rats, and is not excessive in rodent. However, the development of endolymphatic hydrops induced by the serial administration of hydrocortisone or dexamethasone with such dosage provides a puzzling problem in the treatment of Meniere's. Of course, the present results do not clarify the effects of steroids on pathological conditions, such as Meniere's disease, but do not support the concept that endolymphatic hydrops can be reduced by glucocorticoid intake. The present results are compatible with those of Silverstein's clinical trial (7).

The limitation of this study is as follows. Samples were only taken at one time-point, 6 h after the last injection. So, it is not clear if the mild hydrops is an acute response or a more chronic imbalance. To investigate the hydrops, we selected light microscopy. So, we couldn't investigate the cellular damage in the inner ear. Recently, intratympanic administration of steroid therapy is performed to treat an acute sensorineural hearing loss. To conclude the efficacy of steroid therapy, we should compare the results between intratympanically and systemically administered steroids. Further studies are needed to conclude that glucocorticoid therapy affects inner ear fluid homeostasis negatively.

## Data Availability Statement

The original contributions presented in the study are included in the article/Supplementary Materials, further inquiries can be directed to the corresponding author/s.

## Ethics Statement

The animal study was reviewed and approved by Kochi Medical School Animal Care and Use Committee (Approval No. B-00094).

## Author Contributions

All authors listed have made a substantial, direct and intellectual contribution to the work, and approved it for publication.

## Conflict of Interest

The authors declare that the research was conducted in the absence of any commercial or financial relationships that could be construed as a potential conflict of interest.
