# Supplementary figures and images for: Effects of Glucocorticoids on the Inner Ear
Source: Front Surg. 2021 Jan 11;7:596383. doi: 10.3389/fsurg.2020.596383 (PMC7831029; doi:10.3389/fsurg.2020.596383)

## Slide 1
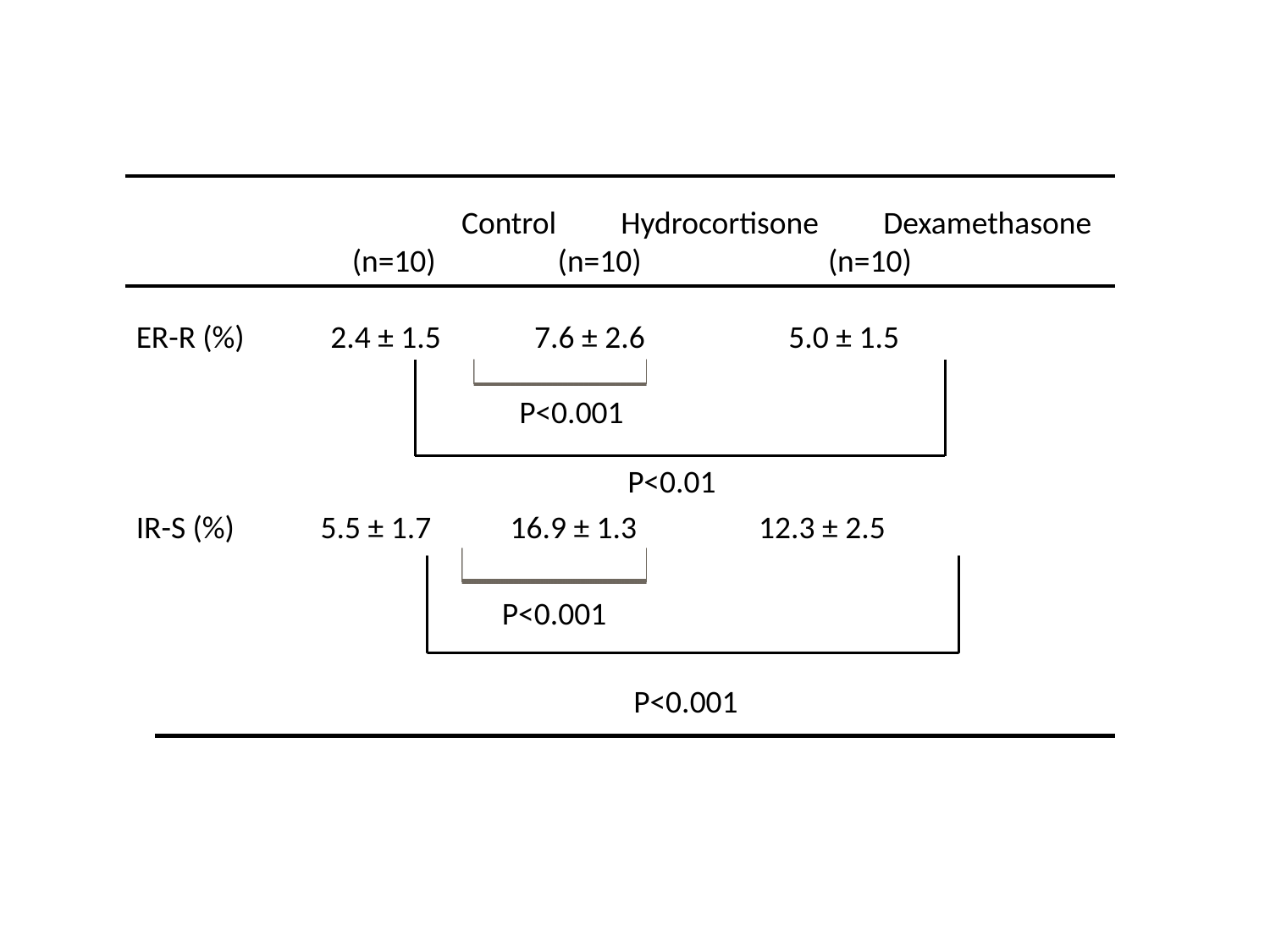

Control Hydrocortisone Dexamethasone
 (n=10) (n=10) (n=10)
ER-R (%) 2.4 ± 1.5 7.6 ± 2.6 5.0 ± 1.5
IR-S (%) 5.5 ± 1.7 16.9 ± 1.3 12.3 ± 2.5
P<0.01
P<0.001
P<0.001
P<0.001

Supplement: Supplementary file 1 [file Presentation_1.PPTX]
